# Nitrogen Application and Rhizosphere Effect Exert Opposite Effects on Key Straw-Decomposing Microorganisms in Straw-Amended Soil

**DOI:** 10.3390/microorganisms12030574

**Published:** 2024-03-13

**Authors:** Yuanzheng Zhao, Shiyu Wang, Meiling Zhang, Li Zeng, Liyu Zhang, Shuyu Huang, Rong Zhang, Wei Zhou, Chao Ai

**Affiliations:** 1 Soil and Fertilizer Institute, Academy of Agricultural and Forestry Sciences, Qinghai University, Xining 810016, China; 2 State Key Laboratory of Efficient Utilization of Arid and Semi-Arid Arable Land in Northern China, The Institute of Agricultural Resources and Regional Planning, Chinese Academy of Agricultural Sciences, Beijing 100081, China; 3 Key Laboratory of Plant Nutrition and Fertilizer, Ministry of Agriculture and Rural Affairs, Beijing 100081, China

**Keywords:** DNA-SIP, key straw-decomposing microorganisms, rhizosphere, straw decomposition

## Abstract

Crop residue decomposition is an important part of the carbon cycle in agricultural ecosystems, and microorganisms are widely recognized as key drivers during this process. However, we still know little about how nitrogen (N) input and rhizosphere effects from the next planting season impact key straw-decomposing microbial communities. Here, we combined amplicon sequencing and DNA-Stable Isotope Probing (DNA-SIP) to explore these effects through a time-series wheat pot experiment with four treatments: ^13^C-labeled maize straw addition with or without N application (S1N1 and S1N0), and no straw addition with or without N application (S0N1 and S0N0). The results showed that straw addition significantly reduced soil microbial alpha diversity in the early stages. Straw addition changed microbial beta diversity and increased absolute abundance in all stages. Growing plants in straw-amended soil further reduced bacterial alpha diversity, weakened straw-induced changes in beta diversity, and reduced bacterial and fungal absolute abundance in later stages. In contrast, N application could only increase the absolute abundance of soil bacteria and fungi while having little effect on alpha and beta diversity. The SIP-based taxonomic analysis of key straw-decomposing bacteria further indicated that the dominant phyla were Actinobacteria and Proteobacteria, with overrepresented genera belonging to *Vicinamibacteraceae* and *Streptomyces*. Key straw-decomposing fungi were dominated by Ascomycota, with overrepresented genera belonging to *Penicillium* and *Aspergillus*. N application significantly increased the absolute abundance of key straw-decomposing microorganisms; however, this increase was reduced by the rhizosphere effect. Overall, our study identified key straw-decomposing microorganisms in straw-amended soil and demonstrated that they exhibited opposite responses to N application and the rhizosphere effect.

## 1. Introduction

The decomposition of plant residues is an important process of the global carbon (C) cycle and a vital link between C stocks in plant and soil systems [1]. In agroecosystems, directly or indirectly returning crop straw to fields is an effective way to accumulate soil organic matter, improve soil structure, enhance biological activity, and balance nutrient cycling [2,3,4]. China produces more than 900 million tons of straw annually [5], including residues from maize (30.50%), rice (27.64%), and wheat (19.21%) [6]. The North China Plain, which is under a wheat–maize rotation system, produces 47% of the total wheat and maize straw in China [7]. This straw provides a rich C source for soil organic C pools in this region. On the other hand, the direct return of large amounts of straw to cropland brings a series of negative effects, such as preventing seedling emergence, facilitating the growth of pests, and contributing to disease [8]. Therefore, it is necessary to further explore the regulatory mechanisms that operate during straw decomposition from the microbial perspective to eliminate these negative effects.

Microorganisms play a crucial role in straw decomposition, and many studies have investigated changes in microbial communities during this process [9,10]. It is well established that microbial-driven straw decomposition is remarkably sensitive to nitrogen (N) nutrients [11]. It is also generally accepted that the amount of N required by microorganisms to decompose straw is much higher than the straw supply capacity during the decomposition process. This causes a flow of N from the soil to microorganisms, limiting the supply of supplements to plants, which is not conducive to crop growth and development. Therefore, N fertilizer is commonly applied with straw return to adjust the C to N ratio (C/N), alleviate N limitation, and regulate nutrient release from the straw [12,13,14]. A global meta-analysis of 334 pairs of data from 53 published articles found that N application could accelerate plant litter decomposition in the early stage [15]. Zhong et al. [14] found that N application could accelerate microbial community assembly during wheat straw decomposition while also increasing the relative abundance of microbial species and functional genes related to C and N cycling. Although N application has a continuous effect on straw-decomposing microorganisms, the mechanism of this impact has not been thoroughly studied in the presence of subsequent crops, especially in crop rotation systems such as the wheat–maize rotation system.

The rhizosphere is an interface that can bridge the gap between plant roots and soil [16]. The biotic and abiotic features of the rhizosphere differ distinctively from those of bulk soil because of the presence of roots. The rhizosphere effect can reduce bacterial diversity and increase bacterial copy number [17]. Plant roots can also recruit bacteria to serve functions such as carbohydrate decomposition, N fixation, and denitrification. Therefore, biogeochemical cycling mediated by microbes within this interface is distinctive [18]. Heredia-Acuña et al. [19] found that the presence of living roots decreased the cumulative decomposition rate of root litter in a greenhouse simulation experiment. However, previous studies of straw decomposition in agricultural ecosystems have paid little attention to this interface. Given the complexity of rhizosphere C decomposers and influencing factors, we speculate that the decomposition of the prior season’s crop residues (e.g., maize straw) in soils will be influenced by the growth of the subsequent season’s crop (e.g., the wheat rhizosphere effect) in a wheat–maize rotation system. Therefore, to better explore key straw-decomposing microorganisms (KSDMs) in actual agricultural production, we should fully consider the rhizosphere effect.

Previous studies have shown that DNA-stable isotope probing (DNA-SIP) is a powerful method for the discovery and characterization of microorganisms utilizing ^13^C-labeled straw C sources [20,21,22]. However, DNA-SIP technology cannot directly distinguish between the KSDMs that utilize ^13^C-straw directly and those that consume ^13^C-intermediates by cross-feeding. In recent years, high-throughput sequencing coupled with heavy and light DNA fractions analysis has been used to mitigate the interference of cross-feeding on the identification of KSDMs that utilize ^13^C-straw directly [10,22,23]. To understand the changes in the structure and composition of key straw-decomposing bacteria and fungi (KSDB and KSDF) in the rhizosphere after N application, we returned ^13^C-labeled straw to the soil and then cultivated wheat in a pot experiment. The soil was divided into rhizosphere and bulk soils, which were harvested on days 7, 14, 30, and 60 after straw return. The objectives of our study were to (i) assess the impacts of N application and the rhizosphere effect on the diversity and abundance of the soil microbial community after straw addition, and (ii) identify the KSDMs and their responses to N application in wheat rhizosphere soils using DNA-SIP.

## 2. Materials and Methods

### 2.1. Preparation of Experimental Materials

Fluvo-aquic soil is the main soil type in the North China Plain, an area that produces large amounts of wheat and maize. So, we choose this type of soil for the pot experiments. The soil was collected from the Modern Agricultural Science and Technology Experimental Demonstration Base, which is affiliated with the Henan Academy of Agricultural Sciences (N 34°47′, E 113°40′), Yuanyang, Henan. The larger impurities in the soil were removed, and the soil was air-dried, ground, sieved (2 mm mesh), and homogenized for the pot experiments. The chemical properties of the fluvo-aquic soil were as follows: SOM, 10.5 g/kg; total N, 0.8 g/kg; available P, 23.8 mg/kg; available K, 92.3 mg/kg; pH, 8.7. The above SOM, total N, and available P were determined using the dichromate oxidation method, Kjeldahl digestion method, and Olsen method, respectively. The available K was measured by ammonium displacement of exchangeable cations. The soil pH was measured with a compound electrode (PE-10, Sartorius, Göttingen, Germany). To prepare the ^13^C labeled maize straw, maize seeds that had been sterilized with 6% H_2_O_2_ were planted in quartz sand that had been pre-sterilized by heating in a muffle furnace at 500 °C for 5 h. Two days after emergence, seedlings of uniform size were transferred into a closed transparent polymethyl room to provide ^13^CO_2_ (^13^C atom% = 98; purchased from Shanghai Institute of Chemical Industry, Shanghai, China). ^13^C labeling was performed for 45 days until harvest. The aboveground part of the maize was then sampled, dried, ground, and sieved. The ^13^C abundance of the labeled maize straw was determined to be 95.05 atom% by isotopic ratio mass spectrometry using an IsoPrime100 system. The total C, total N, and C to N ratio (C/N) of the labeled maize straw were 374.40 mg/g, 19.49 mg/g, and 19.2, respectively. The wheat variety used for the subsequent pot experiments was Longchun 22.

### 2.2. Experimental Design

**Figure 1:**
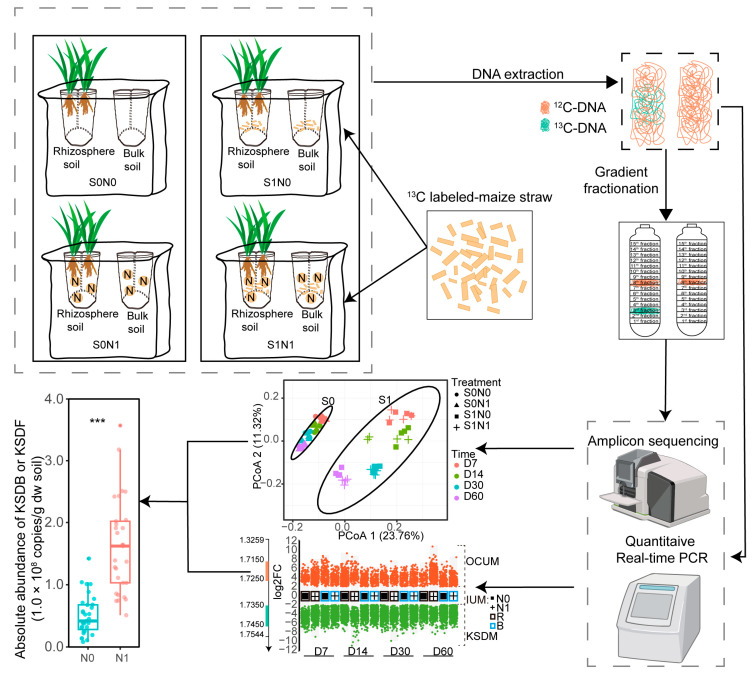
Schematic diagram of potting device and experimental flow diagram. S1N1 and S1N0 indicate straw addition with or without N application. S0N1 and S0N0 indicate no straw addition with or without N application. R and B indicate rhizosphere and bulk soil, respectively. D7, D14, D30, and D60 indicate days 7, 14, 30, and 60 after the start of this experiment, respectively. KSDM represents key straw-decomposing microorganisms, OCUM represents other carbon-utilizing microorganisms, and IUM represents intermediates-utilizing microorganisms. Asterisks represent significant differences in absolute abundance of KSDB or KSDF as evaluated by paired *t*-test (*** *p* < 0.001).

Each culture pot used for the wheat experiment (Figure 1) could accommodate 600 g of soil and two nylon mesh bags (size: 7 cm × 14 cm, 20 μm pore diameter) filled with 20 g of soil. One nylon mesh bag, in which the plants grew, was defined as the rhizosphere soil bag, while another nylon mesh bag that did not contain plants was defined as the bulk soil bag. The purpose of the fine nylon mesh was to separate the rhizosphere and non-rhizosphere compartments and to prevent root contact while allowing water, solutes, and microbes to move between compartments.

The experiment included the following four treatments of 16 replicates (64 pots in total): S0N0 (no straw addition without N application), S0N1 (no straw addition with N application), S1N0 (straw addition without N application), and S1N1 (straw addition with N application). Three wheat plants were sown per replicate. For the straw addition group (S1N0 and S1N1), 0.5% straw (equivalent to dry soil) was added to the soil in the culture pot (^12^C-unlabeled straw) and nylon mesh bags (^13^C-labeled straw), which was equivalent to the straw return amount in the field (8000 kg). For the N application group (S0N1 and S1N1), N was applied at 0.15 g N/kg soil in the form of a urea solution, which was equivalent to 240 kg/ha in the field.

Wheat seedlings were transplanted into the rhizosphere soil bags. After sowing, the culture pots were placed in random order into one of two artificial climate incubators according to whether ^13^C-labeled straw was added. The culture conditions were set as follows: light 16 h, temperature 23 °C, air relative humidity 55%; darkness 8 h, temperature 18 °C, air relative humidity 60%. During this experiment, the soil quantitative moisture was maintained at 40–60% of the maximum water-holding capacity.

Destructive sampling of the wheat pots was conducted on days 7, 14, 30, and 60. Specifically, wheat roots were gently removed from the rhizosphere soil bag, after which the collected soil was regarded as rhizosphere soil while all of the soil in the bulk soil bag was collected as bulk soil. After passing through a 2 mm sterilized sieve, the soils were immediately stored at −80 °C until subsequent DNA extraction.

### 2.3. DNA Extraction and qPCR

Soil DNA was extracted using a soil DNA extraction kit (Fast DNA SPIN Kit for soil, MP Biomedicals, Solon, OH, USA), according to the manufacturer’s instructions. The DNA quality and concentration were determined spectrophotometrically (Nanodrop, PeqLab Biotechnologie GmbH, Erlangen, Germany), and the samples were stored at −20 °C for backup. Real-time fluorescence quantitative PCR (qPCR) was used to quantify the number of bacterial 16S rRNA gene copies and fungal ITS sequences present in the rhizosphere and bulk soil. For a detailed description, please refer to the Supplementary Method. We calculated the absolute abundance of KSDMs by integrating the total copy number detected by qPCR to the sequencing data as follows: absolute abundance of KSDM (copies per g soil) = relative abundance of KSDM (%) × total gene copies of a given sample (copies per g soil).

### 2.4. DNA-SIP

During the subsequent DNA-SIP processes, including DNA fractionation, purification, and qPCR, the non-straw addition groups (S0N0 and S0N1) were used as a control and the straw addition groups (S1N0 and S1N1) were regarded as the experimental group to distinguish heavy and light DNA. DNA-SIP was conducted as previously described [24]. Briefly, the concentration of each DNA sample was determined using a NanoDrop system, after which 3 μg of DNA was thoroughly mixed with CsCl gradients to achieve an initial buoyant density of 1.725 g ml^−1^ using a gradient buffer (GB; 0.1M Tris-HCL (pH = 8), 0.1 M KCl, 1.0 mM EDTA). The mixtures were then transferred to a 5.1 mL centrifuge tube and ultra-centrifuged under the following conditions using a vertical rotor centrifuge (Vti65.2): 44 h, 20 °C, 45 krpm (190,000× *g*), time: Hold, Accel: 9; Decel: no break. Next, sterile water was injected into the top of the tube at a flow rate of 340 µL/min using a NE-1000 fixed flow rate pump (New Era Pump System, Inc., Farmingdale, NY, USA) and 15 equal volume DNA fractionations with different densities were collected from the bottom of the tube. The buoyant density of each collected fraction was subsequently determined using a digital refractometer (AR200, Reichert, Depew, NY, USA). The recovery of nucleic acids from the CsCl salts was conducted by precipitation in two volumes of 30% PEG-6000 (1.6 M NaCl). The recovered DNA precipitates were washed twice with 70% ethanol and dissolved in 30 µL TE buffer, after which the proportions of 16S rRNA and ITS sequences in each DNA fraction were determined by qPCR.

### 2.5. Amplicon Sequence and Bioinformatics Analysis

The ^13^C-labeled heavy and unlabeled light DNA of the rhizosphere and bulk soils were separated by ultracentrifugation, after which the V3-V4 region of bacterial 16S rRNA and the ITS1 region of fungi were amplified by conventional PCR using the 338F/806R and ITS1F/ITS2R primer, respectively, and were then sequenced [24]. For the PCR, a 25 µL reaction mixture composed of 12.5 µL 2 × EasyTaq PCR SuperMix (TransGen Biotech, Beijing, China), 2.5 µL of each forward and reverse primers (10 µM), 1 µL of 10-fold diluted DNA template, and 9 µL ddH_2_O was subjected to the reaction conditions described below. To differentiate each sample, an 8 bp barcode sequence was added to the front of the forward primer. The reaction conditions for the 16S rRNA gene were: pre-denaturation at 94 °C for 3 min followed by 26 cycles of 94 °C for 30 s, 55 °C for 30 s, and 72 °C for 90 s and then extension at 72 °C for 3 min. The reaction conditions for the ITS sequence were: 94 °C for 3 min followed by 28 cycles of 94 °C for 30 s, 54 °C for 30 s, and 72 °C for 90 s and then extension at 72 °C for 3 min. Each sample had two technical PCR replicates, and the products were detected by 2% agarose gel electrophoresis. The two repeated PCR products were mixed and purified, after which the concentrations of purified products were determined using a Quant-iT PicoGreen dsDNA Assay kit (Thermo Fisher Scientific, Waltham, MA, USA). Equal molar mixtures of each sample were then used to create a library for sequencing by the NovaSeq 6000 platform.

QIIME v.1.9.1 [25], QIIME2-2020.8 [26], USEARCH v.11.0 [27], VSEARCH v.2.12.0 [28], and mothur v.1.40.4 [29] were used to process the sequencing data using the method described by Zhang et al. [24]. Briefly, the raw paired-end sequencing data were checked for quality using FastQC v.0.10.1 (https://www.bioinformatics.babraham.ac.uk/projects/fastqc/, accessed on 12 February 2023), then processed as follows: join paired-end (-fastq_mergepairs), extract barcodes (extract_barcodes.py), and demultiplex paired-end fastq (demux). The representative sequences were obtained by the USEARCH and VSEARCH pipelines based on the merged sequences using the following commands: remove primers (-fastx_filter), find non-redundancy reads (-derep_fulllength), cluster unique reads (-cluster_size), and remove chimeric sequences (-uchime3_denovo). Subsequently, all sequences were clustered using a cutoff of 97% similarity to obtain the operational taxonomic units (OTUs) table (-usearch_global). To obtain taxonomic information, the bacterial and fungal OTUs were checked against the SILVA 138 [30] and UNITE [31] databases, respectively, using the classify.seqs command in mothur v.1.40.4 [29]. Additionally, the OTUs defined as chloroplasts, mitochondria, eukaryotes, cyanobacteria, cyanobacteria, angiosperms, and protists were removed. The OTU table was normalized according to the minimum total number of reads across all samples to ensure an even sampling depth for subsequent bioinformatic analysis. The raw amplicon sequence data were deposited in the Sequence Read Archive under BioProject PRJNA942014.

### 2.6. Statistical Analysis

All data were analyzed using SPSS 26.0 (SPSS, Inc., Chicago, IL, USA) except as indicated below. Differences among the groups were identified by one-way ANOVA followed by Tukey’s honest significant difference (HSD) test (*p* < 0.05). Paired-Samples *t*-tests were implemented at a 95% confidence interval. The alpha (Shannon index) and beta diversity of the microbial communities were characterized using QIIME2-2020.8 [26] and visualized using the R software (V3.5.3). To determine the impacts of straw addition, N application, and time on the Bray–Curtis dissimilarities, variance partition analysis (VPA) was performed with the R software (V3.5.3) using the vegan package (V2.6-4). The KSDMs and OCUMs were significantly enriched in heavy DNA and light DNA, respectively. The other microorganisms have been regarded as IUMs. All of them were identified based on DESeq variance analysis using the DESeq2 package (V1.42.1) in R at the OTU level [32].

## 3. Results

### 3.1. Changes of Microbial Alpha Diversity in Straw-Amended Soil

**Figure 2:**
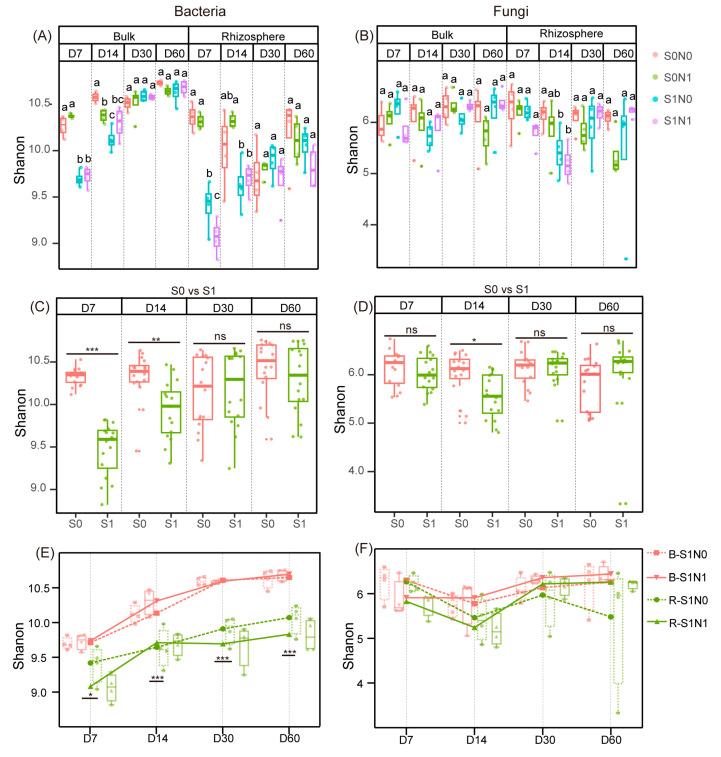
Shannon index of bacteria and fungi (**A**,**B**). Effects of straw addition on Shannon index of bacteria and fungi (**C**,**D**). Impact of N application and rhizosphere effect on Shannon index of bacteria and fungi in straw-amended soils (**E**,**F**). Asterisks represent significant differences in Shannon index as evaluated by paired *t*-test (*** *p* < 0.001. ** *p* < 0.01. * *p* < 0.05. ns, not significant). S1N1 and S1N0 indicate straw addition with or without N application. S0N1 and S0N0 indicate no straw addition with or without N application. R and B indicate rhizosphere and bulk soil, respectively. D7–60 indicate sampling time. Lowercase letters “a–c” represent significant differences in Shannon index as evaluated by one-way ANOVA followed by Tukey’s honest significant difference (HSD) test (*p* < 0.05).

The alpha diversity index (Shannon) of the bacterial and fungal communities in the rhizosphere and bulk soils was calculated for each of the four treatments (Figure 2A,B). Straw addition significantly reduced the soil bacterial Shannon diversity in D7 and D14, but this straw-induced decrease was only obvious for fungi in D14 (Figure 2C,D). Moreover, the presence of wheat roots in straw-amended soil significantly reduced bacterial alpha diversity across all stages, but had no obvious effect on fungi (Figure 2E,F). N application decreased the bacterial Shannon diversity of D7 in the rhizosphere soil, whereas it showed little effect on fungi and bacteria in the rhizosphere and bulk soil in other stages (Figure 2A,E,F).

### 3.2. Shifts in Beta Diversity of Soil Microorganisms after Straw Addition

**Figure 3:**
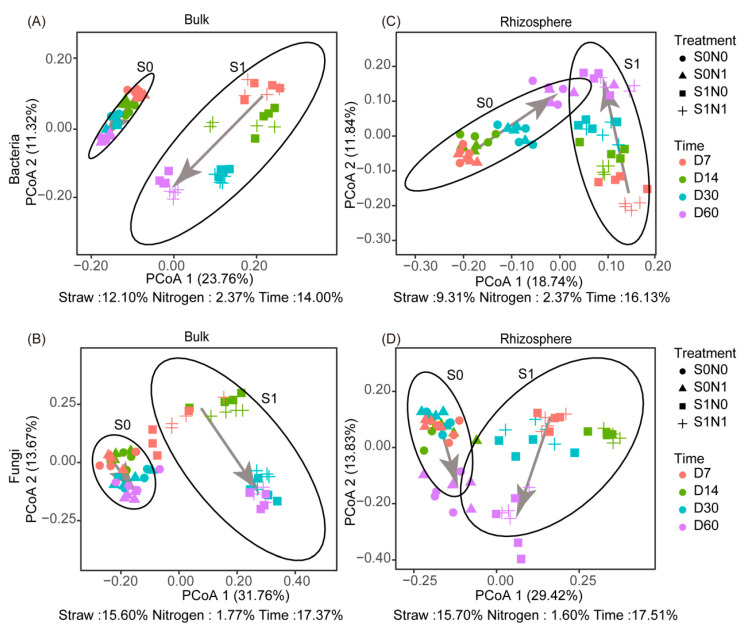
Principal Co-ordinates Analysis of bacterial and fungal community structures in bulk soil (**A**,**B**) and rhizosphere soil (**C**,**D**). Variance partition analysis (VPA) showed the explanation degree of straw addition, N application, and time to bacterial and fungal beta diversity based on the Bray–Curtis distance. S1N1 and S1N0 indicate straw addition with or without N application. S0N1 and S0N0 indicate no straw addition with or without N application. D7–60 indicates sampling time. Arrows represent the succession of microbial community structure over time.

A Principal Co-ordinates Analysis (PCoA) based on the Bray–Curtis distances showed that straw addition dramatically influenced bacterial and fungal community structure, with straw addition being responsible for 9.31% to 15.70% of the community structure changes (Figure 3A–D). This straw-induced distinction persisted until D60 for bacteria and fungi in bulk soils. However, as wheat grew, the rhizosphere effect gradually diminished this straw-induced distinction, causing them to shift in the same direction as soil without straw. N application had little effect on the microbial community structure, explaining only 1.60–2.37% of the changes in community structure.

### 3.3. Succession Pattern of Taxa Composition and Absolute Abundance

**Figure 4:**
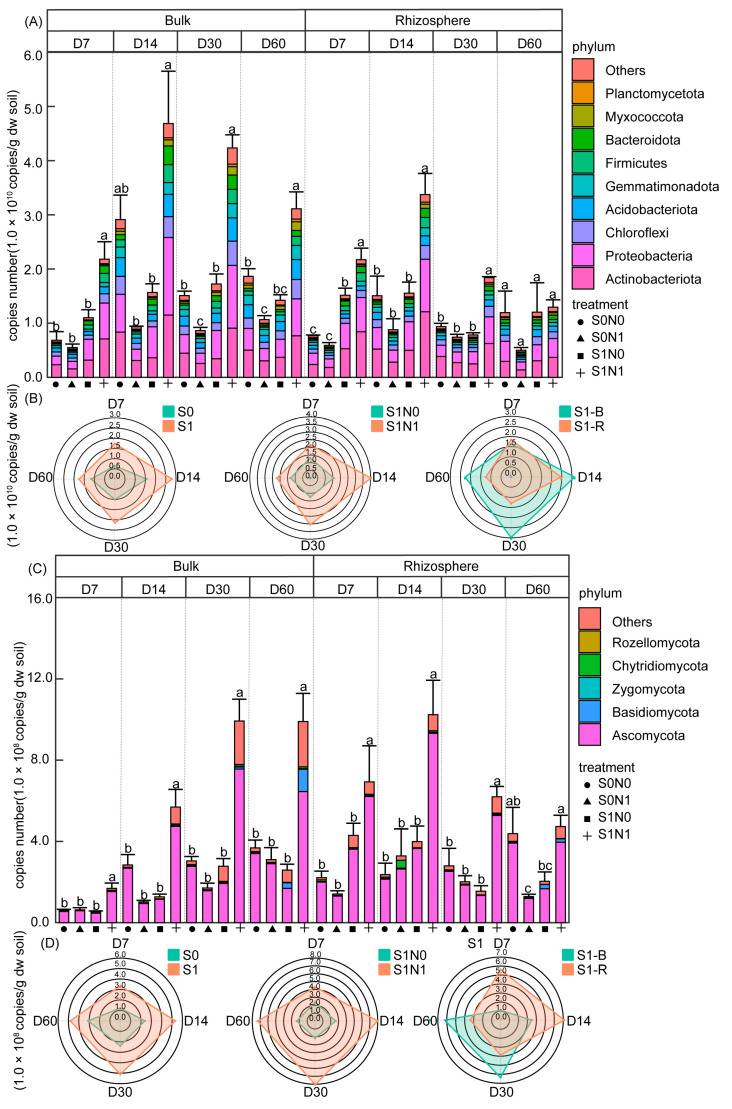
Histograms display changes in the absolute abundance of bacteria and fungi at the phylum level (**A**,**C**). Lowercase letters represent significant differences in absolute abundance between treatments (*p* < 0.05). Left radargrams display the effects of straw addition on soil bacterial and fungal absolute abundance, while the right two show N application and rhizosphere effects on bacterial and fungal absolute abundance in straw-amended soils (**B**,**D**). S1N1 and S1N0 indicate straw addition with or without N application, respectively. S0N1 and S0N0 indicate no straw addition with or without N application, respectively. R and B indicate rhizosphere and bulk soil, respectively. D7–60 indicate sampling time.

The copy numbers of the 16S rRNA gene and ITS rRNA sequence, as well as the top bacterial and fungal phyla, were determined by high-throughput sequencing combined with qPCR. The copies of bacterial 16S rRNA ranged from 5.00 × 10^9^ to 3.37 × 10^10^ and from 5.50 × 10^9^ to 4.69 × 10^10^ copies/g dw soil in rhizosphere and bulk soil, respectively (Figure 4A,B). The copies of the fungal ITS sequence ranged from 1.29 × 10^8^ to 1.02 × 10^9^ and from 5.18 × 10^7^ to 1.40 × 10^9^ copies/g dw soil in rhizosphere and bulk soils, respectively (Figure 4C,D). Temporally, the absolute abundance of fungi showed a continuous increase over time in bulk soil, while bacteria first increased, then decreased, peaking on D14.

Overall, straw addition raised the microbial absolute abundance, especially that of the bacterial phyla Actinobacteria, Proteobacteria, Chloroflexi, Firmicutes, and Bacteroidota and the fungal phylum Ascomycota (Figure 4A,C and Tables S3 and S4). An evaluation of the straw-amended soils revealed that N application significantly increased the absolute abundance of both bacteria and fungi (Figure 4B,D). However, the rhizosphere had different effects on the absolute abundance of bacteria and fungi. Specifically, there were no obvious differences at D7 for bacteria, while a significant rhizosphere-induced reduction was detected at D14–60 (Figure 4B), especially for the phyla Acidobacteriota and Gemmatimonadota (Figure 4A). The growth of wheat led to an initial increase (D7–14) and then a decrease (D30–60) in the absolute abundance of fungi in the straw-amened soils (Figure 4D).

### 3.4. Influence of N Application and Rhizosphere Effect on Key Straw-Decomposing Microorganisms Identified by DNA-SIP

**Figure 5:**
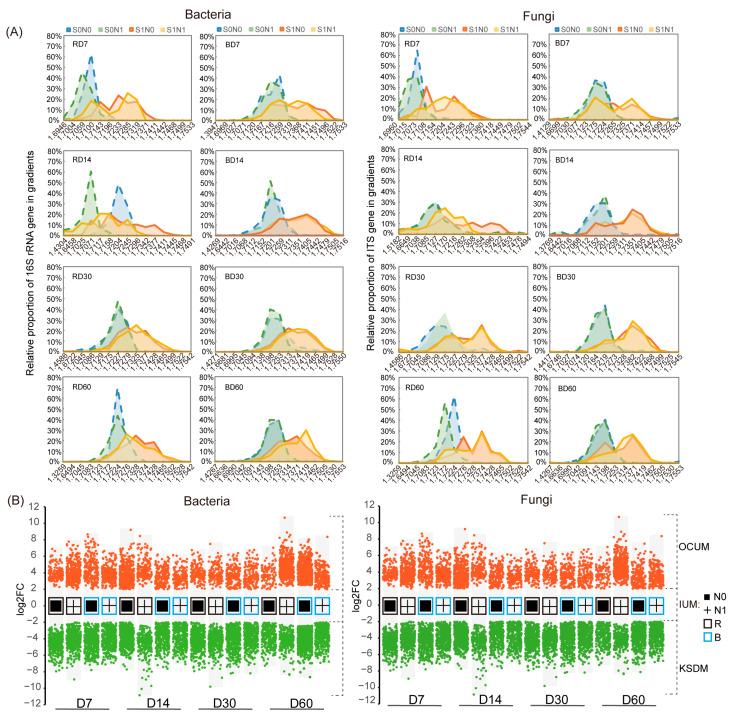
Line graphs represent the distributions of bacterial 16S rRNA gene and fungal ITS sequences based on relative proportion across 15 DNA fractionations (**A**). Column scatter plots represent KSDMs, IUMs, and OCUMs identified by difference analysis between heavy and light groups at the OTU level (**B**). S0N1 and S0N0 indicate no straw addition with or without N application. KSDM represents key straw-decomposing microorganisms, OCUM represents other carbon-utilizing microorganisms, and IUM represents intermediate-utilizing microorganisms. R and B indicate rhizosphere and bulk soil, respectively. D7–60 indicate sampling time.

We used DNA-SIP to identify straw-decomposing microorganisms and then evaluated them based on the difference in bacterial 16S rRNA gene and fungal ITS sequence distribution in each DNA fraction between soils with and without ^13^C-labelled straw (Figure 5A). The fractions with buoyancy densities of 1.7345–1.7450 g/mL in straw-amended soils were considered to be heavy DNA and to represent assimilators of the ^13^C maize straw (directly or indirectly). The fractions with buoyancy densities of 1.7145–1.7250 g/mL were considered to be light DNA and to represent assimilators of the original ^12^C organic matter.

**Figure 6:**
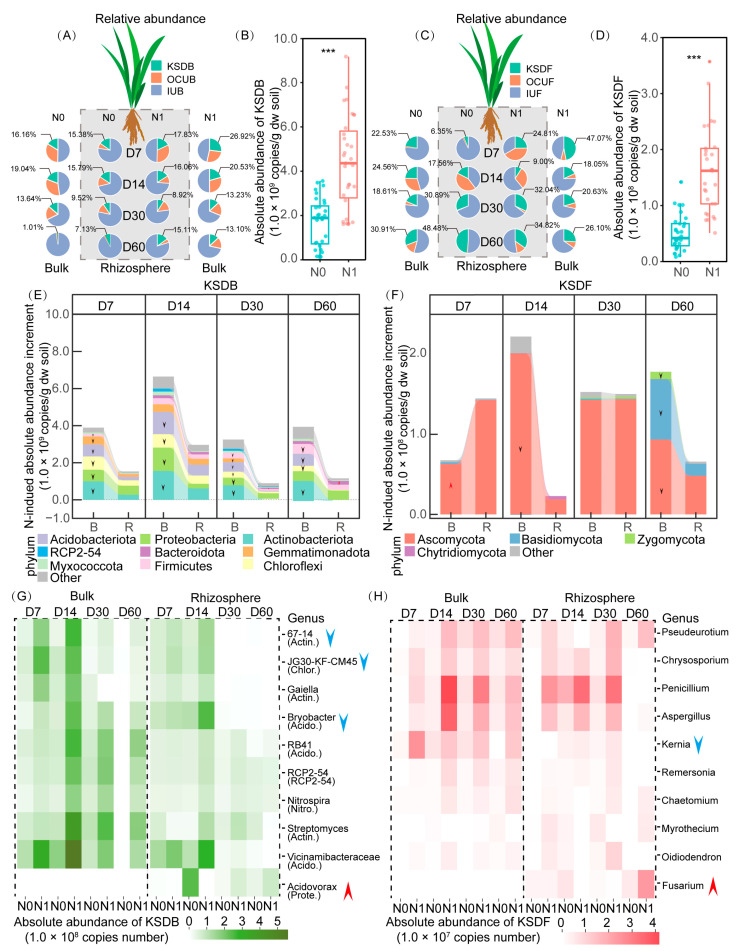
Pie charts show the relative abundance of KSDB and KSDF (**A**,**C**). Box plots display the effect of N application on the absolute abundance of KSDB and KSDF (**B**,**D**). Histograms display the difference in the N-induced increases in KSDB and KSDF between the rhizosphere and bulk soil (**E**,**F**). Heatmaps show the temporal succession in the absolute abundance of KSDB and KSDF at the genus level (**G**,**H**). D7–60 indicate sampling time. Asterisks represent significant differences in absolute abundance of KSDB or KSDF as evaluated by paired *t*-test (*** *p* < 0.001). The upward arrow represents a significant increase, and the downward arrow represents a significant decrease.

To eliminate interference from cross-feeding, we performed a DESeq variance analysis of 29,990 bacterial OTUs and 5163 fungal OTUs based on the negative binomial distribution between heavy DNA and the corresponding light DNA (Figure 5B). This was performed to identify KSDMs, which can directly utilize straw carbon. These organisms primarily belonged to the bacterial phyla Actinobacteriota, Proteobacteria, and Chloroflexi and the fungal phylum Ascomycota (Figure S1), in agreement with the enriched taxa determined by the comparison of absolute abundance (Figure 4A,C). *Streptomyces* and *Vicinamibacteraceae* were the most abundant genera of KSDB, while *Penicillium* and *Aspergillus* were the most abundant genera of KSDF (Figure 6G,H).

Generally, the relative abundance of KSDB decreased over time, while the relative abundance of KSDF oscillated upward over time, regardless of N application or the presence of plant roots (Figure 6A,C). Additionally, the calculation of the absolute abundance of KSDB and KSDF revealed that the N application boosted the absolute abundance of KSDB and KSDF simultaneously (Figure 6B,D and Figure S1). Moreover, this N-indued increment was sharply influenced by the rhizosphere effect (Figure 6E,F). Specifically, the rhizosphere effect reduced the absolute abundance of KSDB in all stages, with the primary reduction being observed in Actinobacteria. Conversely, the rhizosphere effect first increased, then decreased the absolute abundance of KSDF, with Ascomycetes and Basidiomycota being significantly affected in the early and late stages, respectively. At higher resolution, the rhizosphere effect increased the absolute abundance of Acidovorax but decreased that of 67-14, JG30-KF-CM45, and Bryobacter (Figure 6G, Table S3). Among fungi, the rhizosphere effect increased the absolute abundance of Fusarium but decreased that of Kernia (Figure 6H, Table S4).

## 4. Discussion

### 4.1. Effect of N Application and Rhizosphere Effect on Microbial Diversity and Taxa Composition in Straw-Amended Soil

Straw addition reduced the soil microbial alpha diversity in the early stages of our experiment (Figure 2C,D). This reduction may have been caused by the rapid degradation of straw containing oxidizable organic matter, which can release organic acid anions that subsequently reduce the soil pH and community alpha diversity [33]. Simultaneously, straw addition can reduce alpha diversity by inducing the growth of microbes that utilize straw [34], thereby inhibiting microorganisms that prefer to utilize complex soil organic matter. We also found that the presence of plant roots further decreased bacterial alpha diversity in straw-amended soil (Figure 2E), which is in accordance with the results of previous studies [18,35]. These changes indicate that the presence of plant roots could further reduce soil pH, which plays an important role in decreasing bacterial alpha diversity [23,36,37]. Another possible explanation for this phenomenon is that plants can recruit bacteria with specific functions according to their physiological needs [38], thereby reducing diversity by filtering out a subset of microorganisms. N application did not have a significant impact on microbial diversity in the straw-amended soil in our study (Figure 2E,F), which is in accordance with the results of previous studies that have shown that incorporating organic matter into soil can alleviate the alterations in the diversity of soil microbes that are induced by chemical fertilizers [39,40,41].

Straw management, crop planting, and fertilization practices all affect the structure of soil microbial communities in agricultural ecosystems [42,43,44]. In our study, straw addition significantly altered bacterial and fungal community beta diversity, which agrees with the results of other studies [33,45,46]. However, the straw-introduced differences were gradually eliminated as the wheat developed (Figure 3C,D). Similar to the results reported by Chen et al. [47], the rhizosphere microbial community structure of wheat moved in the same direction during growth and development. Numerous studies have attributed this phenomenon to the strong influence of plant roots on the process of bacterial community assembly [48,49]. Plant roots can selectively alter the structure of N-cycling microorganisms in straw-amended soils [18], and this phenomenon in the rhizosphere is closely related to the functional requirements of the plant itself [50]. N application did not have a marked effect on microbial beta diversity in the straw-amended soils. This may have been because of the minor effects of N addition on soil microbial beta diversity, as reported by Wang et al. [51] in a large-scale meta-analysis.

In our study, the presence of straw significantly increased the absolute abundance of soil microorganisms, which was further enhanced by N application in all stages (Figure 4B,D). It is well known that the microbial demand for N is greater than the supply capacity of straw during the microbial reproduction process when straw is used as the carbon source, but that this N requirement is fully met after N application [52]. However, in the present study, the rhizosphere effect had a temporal influence on absolute abundance, where there was an initial increase in fungal absolute abundance that subsequently decreased significantly (Figure 4D). This phenomenon may have occurred because of an increase in root competitiveness for nutrients during the rapid growing period that led to a preferential supply of soil nutrients to plants rather than to soil microorganisms and inhibited the reproduction of soil microorganisms [53].

### 4.2. KSDMs and Their Change in Absolute Abundance in Response to N Application and Rhizosphere Effects

Although a large diversity of microorganisms exists in soil, there are usually only a few that can directly utilize carbon from straw [20]. In this study, these microbes were termed key straw-decomposing microorganisms (KSDMs). To identify the KSDMs and explore their absolute abundance in response to N application and the wheat rhizosphere effect, DNA-SIP combined with high-throughput sequencing was conducted.

*Streptomyces* and *Vicinamibacteraceae* were the primary genera of KSDB, while *Penicillium* and *Aspergillus* were the most abundant genera of KSDF. *Streptomyces* has been reported to have strong capabilities for cellulose and hemicellulose decomposition [54], as well as nitrogen-fixing [55] and antibiotic synthesis [56]. *Vicinamibacteraceae*, the first family within the sd6 Acidobacteria [57] which has been found to contain butyrogenic degradation-related genes and to prefer to utilize complex organic compounds [58], was found to be one of the most abundant microorganisms in the area surrounding our sample sites [59]. *Penicillium* and *Aspergillus* were found to play a role in straw degradation in previous ^13^C-labeled straw decomposition experiments [22], and microbial physiological studies have revealed that they have cellulolytic enzymes and xylanase synthesis systems. In addition, *Aspergillus* can secrete organic acids to increase the availability of soil phosphorus [60].

Not surprisingly, the absolute abundance of KSDB and KSDF (directly utilizing straw-derived carbon microorganisms) was significantly increased by N application. It is well known that crop straw is a high-carbon and low-N organic complex, especially graminaceous crop straw which has a C to N ratio (C/N) that is much higher than those typically found in bacterial (3–12) and fungal (3–60) biomass [61]. Therefore, when microorganisms decompose straw, their biomass C/N will increase, which is not conducive to the maintenance of their stoichiometric ratio. The addition of N can effectively alleviate microbial N limitation, while it has been shown that straw return with N fertilizer can increase the abundance of microorganisms carrying functional genes for carbohydrate decomposition [14], which agrees with our findings. This N-indued increment of KSDMs will promote straw-derived organic matter and nutrient release by enhancing microbial turnover pathways [62], improving soil nutrient availability, and facilitating crop growth and development.

During the wheat–maize crop rotation in the North China Plain, there is a close interaction between the straw decomposition process of the previous crop and the growth and development of subsequent crops. As straw releases nutrients to supply plant growth and development, plants also influence microorganisms that participate in straw decomposition, especially through rhizosphere effects. In this study, we found that the rhizosphere effect reduced the N-indued increase in the absolute abundance of KSDB and KSDF (Figure 6E,F). This reduction may be attributed to the absorption of N nutrients by plant roots. At the phylum level, this effect mainly reduced the abundance of Actinobacteriota, while at the genus level, the absolute abundance of *67-14*, *JG30-KF-CM45*, and *Bryobacter* was decreased. This may have occurred because of the specificity of these genera for the utilization of complex carbon sources. Therefore, when wheat roots were present and developed to later stages, root exudates became the main nutritional source and substrate in the rhizosphere soil, causing the abundance of these microorganisms to decrease as a result of competition. These findings are similar to those of a study conducted by Fu et al. [23]. In addition, the rhizosphere effect led to an enrichment of the *Fusarium* genus, which is a primary pathogen responsible for causing root rot in wheat [63]. The above root-indued changes in KSDMs indicate that the presence of plant roots has a negative effect on straw decomposition [19,64,65] and that straw return may also cause some plant root diseases [66].

## 5. Conclusions

This study used amplicon sequencing, qPCR, and DNA-SIP techniques to identify the key straw-decomposing microorganisms in straw-amended soil and explore their responses to N application and rhizosphere effects. We found that N application and rhizosphere effects exerted opposite effects on straw-related microorganisms. In straw-amended soil, N application increased the absolute abundance of total microorganisms and KSDMs but had little effect on microbial alpha and beta diversity. In contrast, rhizosphere effects decreased bacterial alpha diversity and the absolute abundance of total microorganisms and KSDMs in the later stages of the experiment. Our findings enhance the current understanding of the effects of the common strategy of returning straw to cropland in conjunction with N addition in agricultural production and highlight the influence of plants on straw decomposition.

## Data Availability

The data presented in the study are deposited in the Sequence Read Archive (SRA) Data repository, accession number PRJNA942014.

## Supplementary Materials

The following supporting information can be downloaded at: https://www.mdpi.com/article/10.3390/microorganisms12030574/s1, Supplementary Method: Real-time fluorescence quantitative PCR. Figure S1: Absolute abundance of key straw-decomposing bacteria and fungi; Table S1: Response of bacterial absolute abundance to straw addition, nitrogen application and rhizosphere effects; Table S2: Response of fungal absolute abundance to straw addition, nitrogen application and rhizosphere effects; Table S3: Rhizosphere effect on top 10 genus of key straw-decomposing bacteria on D30 and D60; Table S4: Rhizosphere effect on top 10 genus of key straw-decomposing fungi on D30 and D60.
