# Supplementary material for: Nitrogen Application and Rhizosphere Effect Exert Opposite Effects on Key Straw-Decomposing Microorganisms in Straw-Amended Soil
Source: Microorganisms. 2024 Mar 13;12(3):574. doi: 10.3390/microorganisms12030574 (PMC10974416; doi:10.3390/microorganisms12030574)
Supplement: Supplementary file 1 [file microorganisms-12-00574-s001.zip › microorganisms-2878194-supplementary.pdf]

Supplementary Method: Real-time fluorescence quantitative PCR (qPCR)

The primers used were bacterial 338F/806R and fungal ITS1F/ITS2R. The 10 μL qPCR amplification system consisted of 5 μL SYBR Green, 0.2 μL Rox, 0.2 μL of each Linker and Reverse primers (10 μM), 1 μL 10-fold diluted DNA template, and 3.4 μL ddH_2O, denaturation at 95°C for 5 min, followed by 40 cycles of 95°C for 15s and 60°C for 60s. The reactions were performed using 384-well plates on an ABI Real-Time 7500 system (Applied Biosystems, Waltham, MA, USA) with 4 replicates per sample.

Standard curves were prepared by overnight incubation of cloned plasmids containing 16S rRNA/ITS genes in liquid LB medium. After extraction and purification of the plasmids according to the steps of kit instructions (MiniBEST Plasmid Purification Kit), the concentration of the plasmids was determined by a Nanodrop and the copy number of the target gene was converted according to the molar constant, and then the plasmids were diluted 10-fold with ddH_2O in 7 serial gradients (10^{-8} to 10^{-2}), with 3 replicates of each gradient, and sterile water was used as a negative control during amplification. The amplification efficiency was controlled to be above 89% with $R^2 > 0.97$.

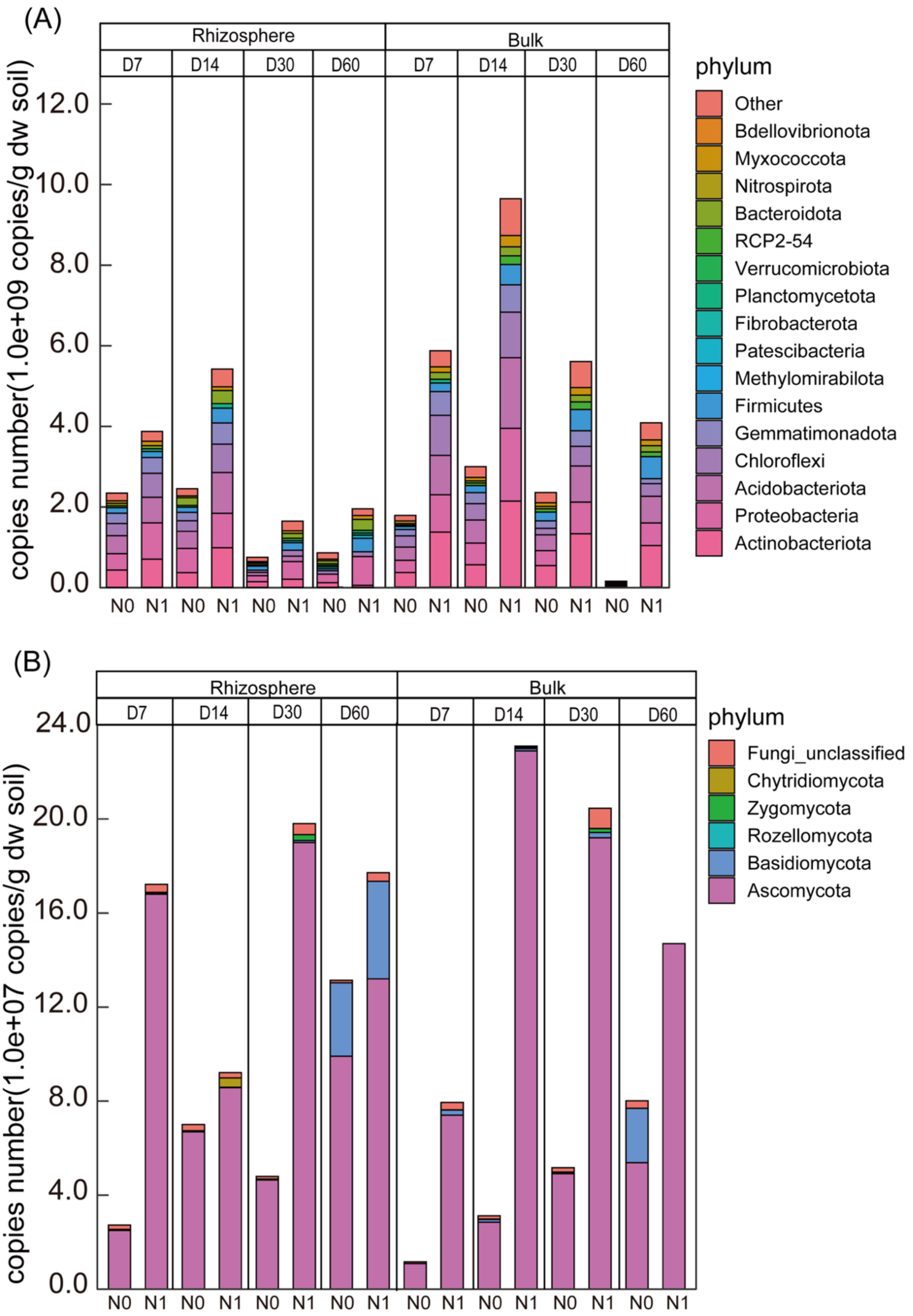

Figure S1. Absolute abundance of key straw-decomposing bacteria and fungi at phylum level (A, B)

Table S1. Response of bacterial absolute abundance to straw addition, nitrogen application and rhizosphere effects. S1N1 and S1N0 indicate straw addition with or without N application. S0N1 and S0N0 indicate no straw addition with or without N application. R and B indicate rhizosphere and bulk soil, respectively

Phylums	S0 vs S1		S1N0 vs S1N1		S1-B vs S1-R	
	t	P value	t	P value	t	P value
Actinobacteriota	-2.98	**0.006**	-4.384	**0.001**	0.534	0.605
Proteobacteria	-3.792	**0.001**	-2.821	**0.014**	1.503	0.169
Chloroflexi	-2.076	**0.047**	-3.178	**0.007**	1.786	0.104
Acidobacteriota	-1.505	0.144	-2.117	0.053	2.809	**0.019**
Gemmatimonadota	-1.402	0.173	-2.623	**0.02**	2.3	**0.044**
Firmicutes	-3.599	**0.001**	-2.98	**0.017**	2.011	0.08
Bacteroidota	-4.041	**0.001**	-2.585	**0.022**	1.532	0.157
Myxococcota	-2.639	**0.013**	-2.15	**0.049**	2.513	**0.031**
Planctomycetota	-2.173	**0.038**	-2.679	**0.018**	1.345	0.214

Table S2. Response of fungal absolute abundance to straw addition, nitrogen application and rhizosphere effects. S1N1 and S1N0 indicate straw addition with or without N application. S0N1 and S0N0 indicate no straw addition with or without N application. R and B indicate rhizosphere and bulk soil, respectively.

Phylums	S0 vs S1		S1N0 vs S1N1		S1-B vs S1-R	
	t	P value	t	P value	t	P value
Ascomycota	-2.469	**0.019**	-3.992	**0.003**	0.753	0.485
Basidiomycota	-1.726	0.095	-0.885	0.402	1.08	0.354
Zygomycota	1.3	0.204	-2.878	**0.012**	0.728	0.494
Chytridiomycota	0.314	0.757	-2.887	**0.012**	2.699	**0.036**
Rozellomycota	-0.122	0.904	-2.393	**0.031**	1.373	0.259

Table S3. Rhizosphere effect on top 10 genus of key straw-decomposing bacteria on D30 and D60

Genus	t	p value
Vicinamibacteraceae_ge	2.239	0.054
Streptomyces	1.459	0.111
Bryobacter	2.37	**0.028**
RCP2-54_ge	1.382	0.128
JG30-KF-CM45_ge	2.502	**0.023**
RB41	1.798	0.061
67-14_ge	2.474	**0.024**
Gaiella	1.53	0.088
Acidovorax	-3.442	**0.007**
Nitrospira	1.236	0.15

Table S4. Rhizosphere effect on top 10 genus of key straw-decomposing fungi on D30 and D60

Genus	t	p value
Pseudeurotium	-0.049	0.481
Chrysosporium	0.381	0.361
Penicillium	0.603	0.284
Aspergillus	1.074	0.156
Kernia	2.399	**0.022**
Remersonia	1.269	0.121
Chaetomium	0.284	0.393
Myrothecium	-1.765	0.056
Oidiodendron	-0.731	0.242
Fusarium	-1.917	**0.044**
